# Systematic characterization of *Gossypium GLN* family genes reveals a potential function of GhGLN1.1a regulates nitrogen use efficiency in cotton

**DOI:** 10.1186/s12870-024-04990-0

**Published:** 2024-04-23

**Authors:** Xiaotong Li, Yunqi Gu, Mirezhatijiang Kayoumu, Noor Muhammad, Xiangru Wang, Huiping Gui, Tong Luo, Qianqian Wang, Xieraili Wumaierjiang, Sijia Ruan, Asif Iqbal, Xiling Zhang, Meizhen Song, Qiang Dong

**Affiliations:** 1 Zhengzhou Research Base, National Key Laboratory of Cotton Bio-Breeding and Integrated Utilization, School of Agricultural Sciences, Zhengzhou University, Zhengzhou, Henan China; 2 Western Agricultural Research Center of Chinese Academy of Agricultural Sciences, Changji, 831100 Xinjiang China; 3 National Engineering Research Center of Cotton Biology Breeding and Industrial Technology /Institute of Cotton Research of CAAS, Anyang, 455000 Henan China; 4 Department of Agriculture, Hazara University, Khyber Pakhtunkhwa, Mansehra, 21120 Pakistan

**Keywords:** *Gossypium*, Glutamine synthetase, Gene family, Gene expression, Nitrogen use efficiency

## Abstract

The enzyme glutamine synthetase (*GLN*) is mainly responsible for the assimilation and reassimilation of nitrogen (N) in higher plants. Although the *GLN* gene has been identified in various plants, there is little information about the *GLN* family in cotton (*Gossypium* spp.). To elucidate the roles of *GLN* genes in cotton, we systematically investigated and characterized the *GLN* gene family across four cotton species (*G. raimondii, G. arboreum, G. hirsutum,* and *G. barbadense*). Our analysis encompassed analysis of members, gene structure, cis-element, intragenomic duplication, and exploration of collinear relationships. Gene duplication analysis indicated that segmental duplication was the primary driving force for the expansion of the *GhGLN* gene family. Transcriptomic and quantitative real-time reverse-transcription PCR (qRT-PCR) analyses indicated that the *GhGLN1.1a* gene is responsive to N induction treatment and several abiotic stresses. The results of virus-induced gene silencing revealed that the accumulation and N use efficiency (NUE) of cotton were affected by the inactivation of *GhGLN1.1a.* This study comprehensively analyzed the *GhGLN* genes in *Gossypium* spp*.*, and provides a new perspective on the functional roles of *GhGLN1.1a* in regulating NUE in cotton.

**Supplementary Information:**

The online version contains supplementary material available at 10.1186/s12870-024-04990-0.

## Introduction

Nitrogen (N) plays a crucial role in plant development and growth, serving as a vital component of nucleic acids, proteins, chlorophyll, and other essential compounds. N levels also influence photosynthesis, carbon assimilation, and the regulation of endogenous hormones. Nitrate (NO_3_^−^) and ammonium (NH_4_^+^) represent significant forms of inorganic N sourced from the environment, yet they cannot be directly utilized as substrates for protein production and the synthesis of various secondary metabolites [1]. Following absorption by plant roots from the soil, NH_4_^+^ or NO_3_^−^ enters the cytoplasm, where it undergoes conversion into the organic N compound glutamine. This transformation occurs through the sequential action of the enzymes glutamine synthetase (GS) and glutamate synthase (GOGAT), collectively known as the GS/GOGAT cycle [2–4]. Therefore, GS is the major enzyme that assimilates N in higher plants.

GS in higher plants belongs to GSII, which can be divided into cytoplasmic GS (GLN1) and plasmid GS (GLN2) related to their subcellular localization [5]. GLN1 is primarily distributed in plant roots and mainly participates in the transport of sources of stored N during seed germination and completing the transfer and reuse of N during the senescence of leaves [6]. *GLN2* is primarily distributed in the leaves and mainly assimilates the ammonia that is released by photorespiration and NO_3_^−^ reduction [7].

Since the cloning and sequencing of the structural gene of the bacterial *GLN* from the cyanobacterium *Anabaena* in 1983, the cDNA and genomic DNA of *GLN* have been cloned in various plants, including barley [8], maize [9], rice [10] and pea [11]. Sakakibara et al. isolated 5 cDNA clones of *GLN* from the maize cDNA library [12]. Sakamoto et al. also isolated and sequenced three cDNA clones of *GLN* from a rice cDNA library [9]. In *A. thaliana*, six *GLN* genes were clearly expressed in specific tissue; for example, *Gln1:1* is highly expressed in epidermal root cells and may play a role in sensing exogenous N and functioning to serve in the primary assimilation of NH_4_^+^ [1]. *Gln1:2* is highly expressed in the leaves, and only *GLN1* is expressed in mesophyll cells. *Gln1:2* encodes an enzyme with a low affinity for NH_4_^+^ that is essential for its detoxification [13, 14]. Furthermore, the *Gln1:3* genes are highly expressed in the roots and stems and involved in the formation of roots [15]. The *GLN2* group exhibits the highest expression levels in green tissue and primarily serves in the re-assimilation of photorespiratory NH_4_^+^ [5, 16]. In maize, *GLN1* is encoded by five nuclear genes. Among them, *GLN1-1* exhibits the highest expression levels, particularly in the roots, whereas *GLN1-2* is predominantly expressed in the stems. *GLN1-3* and *GLN1-4* show significant expression in the leaves. Specifically, *GLN1-3* influences panicle number, while *GLN1-4* governs grain weight regulation. Nevertheless, *GLN1-5* was moderately expressed across all the tissues and organs [17]. In barley, *GLN1* is encoded by three nuclear genes, and *HvGS1-1* is highly expressed in vascular tissues of different organs. The *HvGs1-2* is highly expressed in the epidermal cells of leaves, while *HvGS1-3* is highly expressed in the ears of wheat [18].

*GLN* has also reported to be involved in the responses to several types of plant stress [19, 20]. Many different plants have been shown to regulate the expression and activity of their GLN isoforms in response to stressors, such as higher levels of N [21], drought [22, 23], cold [24, 25], salinity [20] and metal toxicity [26, 27]. Many studies have explored the *GLN* enzyme, and some hypothesized that it improves tolerance to stress. For example, a study that compared the levels of expression and activities of different *GLN* genes in rice under drought stress showed that a significantly maintained level of *OsGS2* and the over-expression of *OsGS1:1* might be contributing factors to the traits for drought resistance in the drought-tolerant rice cultivar Khitish [28]. Additionally, studies that compared gene expression examined various genotypes of durum wheat under salt and drought stress revealed that the most resilient genotype had the highest activity of *GLN* and increased levels of expression of *GLN1* and *GLN2* under stress conditions compared with the control plants [23]. Nagy et al. found that compared with the sensitive cultivars, the drought-tolerant wheat cultivars had increased *GLN* activity in the flag leaves [29]. Lothier observed that *AtGLN1.2* was significantly expressed in the root and leaf tissues and the mesophyll cells of aging leaf tissues of *A. thaliana* under high N conditions [30].

Cotton (*Gossypium* spp.) is a significant economic crop that provides 35% of the natural fiber used throughout the world. Cotton requires higher levels of N for proper growth and development, and a lack of N in the soil may reduce the growth, yield, and high-quality fiber production of cotton. Therefore, it is critical to comprehend the molecular mechanisms of N use in cotton. The role of *GLN* family genes has been defined in various plants, however, little is known about the function of the *GLN* gene family in cotton. We examined the entire *GLN* gene family of four species, including two diploid species (*G. raimondii* and *G. arboreum*), and two tetraploid species (*G. hirsutum* and *G. barbadense*) to clarify the functions of cotton *GLN* genes. This study thoroughly investigated the phylogenetic distribution, chromosomal position, gene structure, preserved motifs, duplication pattern, and selective stress stimuli of the cotton *GLN* genes. In addition, the profiles of expression of the *GhGLN* genes were evaluated using the data from quantitative real-time reverse-transcription PCR (qRT-PCR). Virus-induced gene silencing (VIGS) was used to evaluate how the genes impacted the accumulation and N use efficiency (NUE) in cotton. The findings of this study provide a basis for the functional characterization of *GhGLN*s.

## Materials and methods

### Identifying identification of the *GLN* Genes

The genomic sequences of the four cotton species [*G. arboreum* (A2; CRI assembly), *G. raimondii* (D5; JGI assembly), *G. barbadense* (AD2; sea-island cotton; HAU assembly), and *G. hirsutum* (AD1; upland cotton; CRI assembly)] obtained from the CottonGen database (http://www.cottongen.org/) were used to identify the *GLN* protein family members based on their homology with the sixGLN proteins from the *A. thaliana* TAIR database (https://www.arabidopsis.org/). We used the *GLN* domain (PLN02284) as a multiple BLAST query against the cotton database to locatecandidate GLN sequences in the genome of the four cotton species using HMMERv 0.3.1. We checked the putative *GLN* members for the presence of fully or partially conserved motifs in more detail using the SMART and CDD databases. The genomic DNA, CDS, protein, and cDNA sequences of the *GLNs* were assembled using the Cotton Gen (http://www.cottongen.org/) and Cotton FGD databases (https://cottonfgd.org/). The search tool of the Ensembl Plants database was used to evaluate the size of the genes, the length of the proteins, and the number of introns (http://plants.ensembl.org/). Additionally, the molecular weight (MW) and isoelectric point (pI) values of the proteins were obtained using the ProtParam program (https://web.expasy.org/protparam). TBtools was used to establish the structure of the *GLN* family genes based on their coding and genomic sequences [31].

### Data retrieval and sequence analysis

The genomic fasta files and annotated gff3 files of *G. arboreum*, *G. hirsutum*, *G. barbadense* and *G. raimondii* were downloaded from the CottonFGD. The genomic sequences of *A. thaliana* were obtained from the TAIR 10 database (https://www.arabidopsis.org/). MEGA 10 was used for the phylogenetic analysis of the *GLN* gene family in the four cotton species and *A. thaliana*. The neighbor-joining (NJ) method was used to create a phylogenetic tree at 1000 bootstrap iterations. The exon/intron structure information of the *GLN* gene family of cotton was obtained from CottonFGD, and the gene structures were visualized graphically using TBtools. The default parameters of the MEME tool were used to identify the conserved motifs of the protein sequences of cotton that contained *GLN*. Annotated gff3 files were used to determine where the *GLN* genes that were identified were located on the chromosomes. The putative regulatory elements were found in the 2000 bp 5'-upstream regions of the *GLN* genes using the default parameters of TBtools. TBtools was also used to extract information and conduct a preliminary regulatory-element analysis. ClustalX 1.83 was used to align the predicted protein sequences.

### Evolutionary analysis of the GLN genes

The homologous *GLN* genes of the four cotton species were identified by contrasting their coding patterns. TBtools was used to retrieve the collinearity pairs from the *GLN* family and generate a collinearity map of the *GLN*s. Similarly, synonymous and nonsynonymous substitution rates (Ks and Ka, respectively) were calculated using TBtools. The Ka/Ks ratio > 1, Ka/Ks ratio = 1 and Ka/Ks ratio 1 indicated positive, neutral, and negative/purifying selection, respectively [32, 33]. Every pair of identical *GLN* genes was subjected to the predicted selection pressure.

### Plant treatments and the qRT-PCR analysis

The experiment was conducted in the greenhouse at the Institute of Cotton Research of the Chinese Academy of Agricultural Science. Healthy seeds (*G. hirsutum L. acc.* TM − 1) were sown in a 1:1 mixture of sand and vermiculite for 1 week in a germinator. After the two cotyledons had fully opened, seedlings of uniform height were selected and transplanted into 8 L plastic containers and grown at 28℃ under a 16/8 h light/dark cycle with 60% relative humidity, as previously reported in our studies [34, 35]. During the first week after transplanting, all the seedlings were supplied with 1/2-strength Hoagland solution, followed by the full strength solution, until the seedlings had reached the three true leaves stage. The Hoagland culture medium was renewed every 5 days.

Based on the literature and previous research, we identified three different N concentrations for plant growth, which included low N (LN; 0.25 mM of the NO_3_^−^ solution), normal N (NN; 5 mM of the NO_3_^−^ solution) and high N (HN; 10 mM of the NO_3_^−^ solution) [36, 37]. After 4 weeks of growth, three plants from each treatment were collected for qRT-PCR analysis. For the N induction treatment experiment, plants with three true leaves were cultured with no-N Hoagland solution for 5 d, and the leaf and root samples were collected at 0 h, 1 h, 3 h, 6 h, 12 h and 24 h after N resupply treatment. Three plants from each treatment were collected for qRT-PCR analysis. The plants were grown under normal N for 2 months to analyze the pattern of expression of the *GhGLNs*. The leaves, roots, stem, pistil, stamen, petals and calycle samples were collected for qRT-PCR analysis.

We used the Real-time PCR (TaqMan) Primer and Probe Design Tool (Real-time PCR Primer Design-Real-time PCR Probe Design [-GenScript Biotech, Piscataway, NJ, USA]) to generate the qPCR-specific primers for the 10 representative *GhGLN1* family genes using the N treatment transcriptome data. Table S5 contains the list of the primer sequences used for the qRT-PCR analysis. We obtained the total RNA using an RNA prep Pure Plant kit (TianGen, Beijing, China) and measured the quantity and quality of the RNA samples with a spectrophotometer. The total RNA was extracted from the leaf samples using an EASYspin plus Plant RNA Kit (Aidlab Biotechnologies Co., Ltd., Beijing, China) and reversed-transcribed into cDNA using a TransStart® Top Green qPCR SuperMix (+ DyeII) [38]. The housekeeping gene, β-actin, served as an internal reference for the qRT-PCR analysis, and the delta-delta Ct (2^−∆∆Ct^) method was utilized to determine the relative levels of expression of the genes [39, 40].

### VIGS treatment and measurement of the concentration nitrogen

To silence the expression of *GhGLN1.1a*, we cloned its 300-bp coding region into the CLCrV vector. Table S5 contains the cloning primer sequences. The created construct, recombinant CLCrV-*GhGLN1.1a*, was then combined in a 1:1 ratio with the helper vector (pCLCrV) strain at an OD600 of 1.5, and the recombinant and empty CLCrV vectors were transferred into *Agrobacterium tumefaciens* GV3101 for agroinfiltration of the plants. The infiltrated plants were kept at a constant temperature of 25 °C to facilitate efficient viral infection and transmission. The plants were grown in a 1:1 mixture of nutrient soil and vermiculite.

The contents of N in the leaf samples of the boll opening of cotton plants were determined using the Kjeldahl method [41]. The dried samples of all the functional leaves were crushed into a fine powder. Approximately 0.2 g of each sample powdered was weighed and digested with sulfuric acid-hydrogen peroxide (H_2_SO_4_-H_2_O_2_), and then analyzed for their N content using the AutoAnalyzer III (AA3; SEAL Analytical, Inc., Mequon, WI, USA). The accumulation of N and the NUE in the plants was calculated as described by Iqbal [36] The accumulation of N was determined as the product of the content of total N in the leaves and their total dry weight. The NUE was measured as the total leaf dry weight divided by the leaf total N content (Table S6). Each measurement was conducted in triplicate, and the mean and standard deviation (SD) of the three replicates was used to obtain the final measurement. A t-test was used to determine the significance of the differences between the samples.

## Results

### Genome-wide characterization and chromosomal position analysis of the *GLN* genes in four cotton species

Members of the GLN protein family were identified in four species of cotton (*G. arboreum*, *G. raimondii*, *G. barbadense* and *G. hirsutum*) based on their similarity to the six GLN proteins from the *A. thaliana* TAIR database. Finally, 42 putative *GLN* genes were obtained from the genomes of the four cotton species, including seven each from *G. raimondii* and *G. arboreum* and 14 each from *G. barbadense* and *G. hirsutum*. The properties of these 42 GLN proteins are shown in Table S1. Moreover, the four cotton species had coding amino acid lengths that ranged from 266 to 441 and molecular weights that ranged from 29.293 and 48.559 kDa. Nine proteins had PI values higher than 7, and these included Ga03G0313.1 (7.521), Gh_A02G031900.1 (7.521), Gh_D02G037300.1 (7.521), Gh_A09G220300.1 (7.9), Gbar_A02G002530.1 (7.521), Gbar_D02G003200.1 (7.521), Gbar_A09G021170.1 (7.856), Gorai.005G035800.1 (7.521) and Gorai.006G218100.1 (7.234). The remaining proteins had pI values below 7, indicated that they were acidic, whereas those whose pI values above 7 were alkaline. Additionally, the grand average of hydropathy (GRAVY) scores of all 42 proteins were negative, which indicated that they were all hydrophilic.

A map of the gene location was created by TBtools to show the distribution of *GLN* genes in the chromosomes of the four cotton species (Figure S1). Each chromosome contained one *GLN* gene, and most of these genes were found near the ends of the chromosomes. Chromosomes 8 and 10 of all four cotton species had no *GLN* genes. There were seven *GLN* genes over all in subgenomes A and D of *G. barbadense*, and seven *GLN* genes in subgenomes A and D of *G. hirsutum*. This indicated significant conservation of the *GLN*s between the A and D genomes of these cotton species.

### Gene structure, phylogenetic analysis and an examination of the conserved motifs

**Fig. 1:**
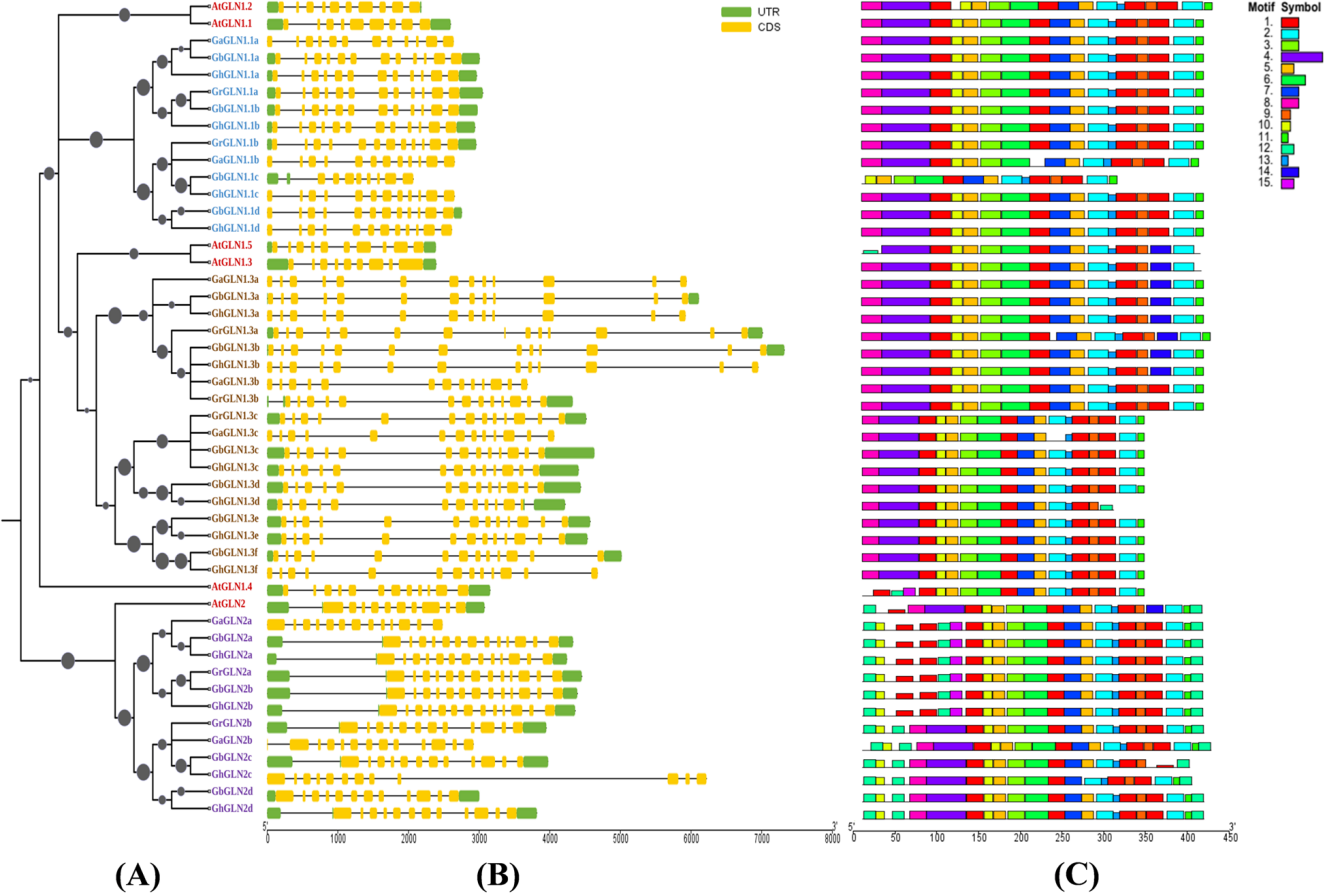
Comprehensive analysis of the *GLN* gene family traits of the four cotton species. **a** Phylogenetic analysis of the *GLN* proteins from four cotton species. A bootstrap analysis with 1000 iterations was performed after the phylogenetic tree was created using the neighbor-joining (NJ) technique. **b** Exon organization of the *GLN* genes. Yellow boxes represent exons. **C** The 15 conserved motifs of the *GLN* proteins of the four cotton species. The motifs are indicated by various colored boxes

We obtained protein sequences from four different cotton species and *A. thaliana* that contained the *GLN* domain to create a phylogenetic tree to clarify the phylogenetic relationships of the *GLN* genes (Fig. 1A). Five *A. thaliana* sequences with the highest similarity with those of cotton were named *AtGLN1.x* (x denotes from 1–5). The *GLN* sequences matched the *AtGLN1.1–5* and *AtGLN2* sequences were denoted *GLN1.1–5* and *GLN2*, respectively.

We analyzed the conserved motifs and the gene structure of the *GLN* gene family in cotton in more detail (Fig. 1b, c)*.* We identified 15 conserved motifs in the four cotton species using the MEME-MEME Suite (https://meme-suite.org/meme/doc/meme.html) and TBtools (Fig. 1C), and the different motifs occurred in varied combinations in the various subfamilies in varied combinations (Figure S2). The gene annotation gff3 files were used to evaluate the structure of the *GLN* genes, and most of the motifs in the *GLN* genes of the four cotton species showed comparable patterns. However, motifs 7, 12 and 13 were only found in the *GLN2* group, while motif 15 was only found in *GbGLN1.1c* and* AtGLN1.2.*

TBtools was utilized to generate exon and intron structure plots, enhancing our comprehension of the diverse structural characteristics of the *GLNs* (Fig. 1B). We discovered that the number of exons/introns, exon length and motif information shared by all the paralogs found in the same branch of the phylogenetic tree were identical, which indicated that there had been no structural and functional changes after the gene pairs had formed.

### Regulatory elements of the *GhGLN1* genes

**Fig. 2:**
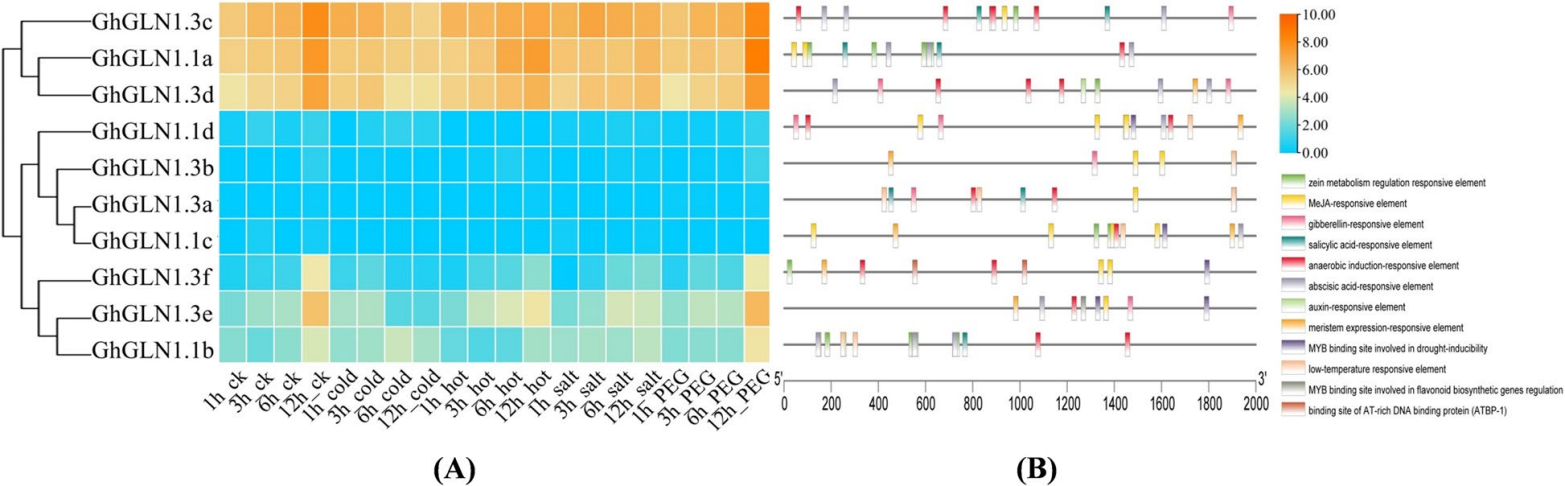
Regulatory elements of the *GhGLN1* genes. **A** Expression levels of 10 *GhGLN1* genes under abiotic stresses. The expression levels are shown as the log2FPKM values. FPKM; Fragments Per Kilobase of transcript per Million mapped reads. **B** The regulatory regions of 10 *GhGLN1* gene members. The regulatory regions are located 2000 bp upstream of ATG. The position "0" in the figure corresponds to the ATG. The color blocks on the lines indicate various regulatory elements

We analyzed the expression levels of the 10 *GhGLN1* genes under abiotic stresses based on the already published RNA-seq data (accession number: PRJNA248163 [42]) and the regulatory components in the 5' upstream regions of *G. hirsutum* to determine the functions of the *GLN* gene family in *G. hirsutum*. *GhGLN1.1 a*, *GhGLN1.3 c* and *GhGLN1.3d* were highly expressed in various organs under abiotic stresses, compared with the other seven genes. *GhGLN1.1 a*, *GhGLN1.3 c* and *GhGLN1.3d* were highest under cold, heat, salt and drought (polyethylene glycol (PEG)-induced) stresses, which indicated that these genes may have some regulatory functions under cold, heat, salt and drought stresses (Fig. 2A). Moreover, *GhGLN1.1 a*, *GhGLN1.3 c* and *GhGLN1.3d* contained many anaerobic induction regulatory elements and phytohormone regulatory elements (abscisic acid, methyl jasmonate (MeJA) and salicylic acid (SA) responsiveness) (Fig. 2B). Moreover, putative regulatory elements were prevalent but not conserved in the 5'-upstream regions of the *GhGLN* family genes of *G. hirsutum* (Fig. 2). Furthermore, only a few *GLN* genes had certain regulatory elements (such as responsiveness to low-temperature), which suggested that some of these genes are triggered by different signals.

### Alignment of the *GhGLN* proteins

Figure S3 shows the identities of the *GhGLN1* and *AtGLN1* proteins and the percentage identity between the translated protein sequences of *GhGLN1* and *AtGLN1*. High degrees of sequence similarity existed between the *GhGLN1* protein sequences and each of the five *AtGLN1* protein sequences. The *GhGLN1* proteins were 80.8% to 90.4% identical to their *AtGLN1* orthologous proteins. Two conserved Pfam domains (Pfam 03951 and Pfam 00120) specific to glutamine synthetase enzymes were found in all the *GhGLN* protein sequences (Figure S4). Based on previous research, some of the ammonium/glutamate-binding pocket residues (shown in short purple lines) were highly conserved [43], and some (shown in boxes) were associated with the characteristics of affinity for NH_4_^+^ [44]. The polar amino acids Q49 and S183 were identified as playing crucial roles in the highly specific binding properties of NH_4_^+^ to *AtGLN1.1* and *AtGL1.4.* While S183 remained strictly conserved in *GhGLN1.3c*, *GhGLN1.3d*, and all *GhGLN1.1* proteins, Q49 was not conserved in all *GhGLN* proteins. In the *GhGLN1* sequences, the polar Q49 was transformed into basic glutamate K49 and R49. It could be that the properties for the affinity for NH_4_^+^ of these modified amino acids were maintained in *GhGLN1.3c*, *GhGLN1.3d* and all the *GhGLN1.1* proteins. However, the residues K49, A183 and E49 found in the low-affinity enzymes *AtGLN1.2, AtGLN1.3* and *AtGLN2* were preserved in most of the *GhGLN1.2*, *GhGLN1.3* and *GhGLN2* protein sequences, which indicated that the low NH_4_^+^ affinity qualities of these three protein classes had not changed (Figure S4).

### Analysis of *GLN* gene duplication and collinearity in the four cotton species

**Fig. 3:**
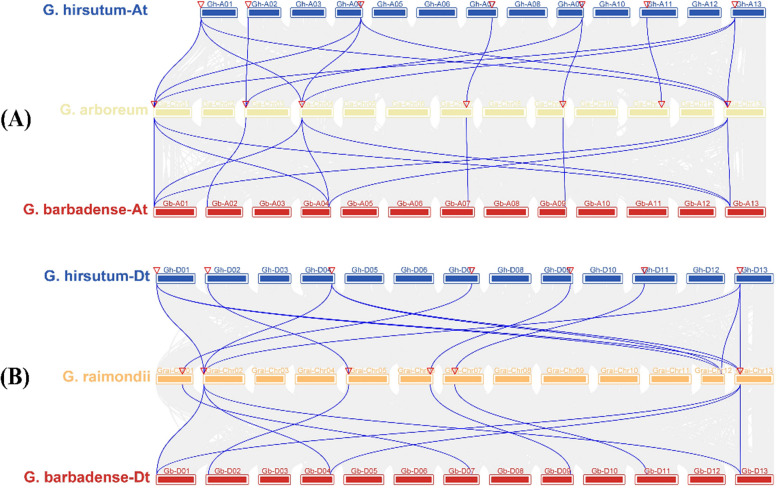
At sub-genomes *GLN* genes collinearity relationship and Dt sub-genomes *GLN* genes collinearity relationship in cotton. **A** Synteny and collinearity relationships between *G. hirsutum* At sub-genomes, *G. barbadense* At sub-genomes and *G. arboretum.* **B** Synteny and collinearity relationships between the *G. hirsutum* Dt sub-genomes, *G. barbadense* Dt sub-genomes and the *G. raimondii*. The collinearity of the *GLN* genes across several genomes is shown by blue lines

We examined the intraspecific duplication events of the *GLN* genes to understand the evolution of the *GLN* gene family in cotton. Gene expansion and the emergence of novel gene functions are often shaped by gene duplication events. In this study, we employed the TBtools MCScan package to conduct a homologous BLAST analysis of cotton amino acids. *G. arboreum* contained 16,143 collinear genes, 1235 collinear blocks and 2375 tandem repeat genes (Table S2). However, *G. raimondii* had 16,642 collinear genes, 707 collinear blocks and 909 tandem repeat genes (Table S2). *G. barbadense* contained 55,672 collinear genes, 3299 collinear blocks and 3304 tandem repeat genes (Table S2), while *G. hirsutum* had 3334 tandem repeat genes, 3242 collinear blocks and 55,454 collinear genes (Table S2). Paralogous pairings of the *GLN* gene families were produced owing to whole segmental duplication or genome duplication. Transpositions might have produced many paralogous genes in the four cotton species, with a minimal contribution from tandem repeat genes. Four scattered and three segment duplicates were discovered in *G. arboreum* (Table S3). Similarly, four genes that had undergone scattered duplications and that which had undergone segmental duplications were identified in *G. raimondii*. In tetraploid cotton species, *G. barbadense* exhibited 14 segmental gene duplications, which was similar to the findings in *G. hirsutum* (Table S3). Homologous BLAST findings demonstrated the collinearity of the four cotton species (Fig. 3). A-Gh At, A-Gb At, D-Gh Dt and D-Gb Dt gene pairs from the four cotton species were used for the collinearity test (Fig. 3). Unexpectedly, there were fewer similarities of duplication between the D genomes of *G. arboreum*, *G. barbadense* and *G. hirsutum* than between their A genomes. This suggested that the A genome contributed more to duplication during the evolutionary cycle, which generated more *GLN* gene family traits.

The Ka/Ks ratio reflects the evolutionary path of a gene or gene area. There were 3, 1, 18 and 21 pairs of gene duplication in *G. arboreum*, *G. raimondii*, *G. barbadense* and *G. hirsutum*, respectively (Figure S5). The *GLN* genes in *G. arboreum* acquired segmental duplication between 7.71 and 55.72 million years ago (Mya), while those in *G. raimondii* were duplicated between 7.26 and 42.29 Mya. Segmental duplication was observed between 0.44 and 26.04 Mya in *G. barbadense* and between 0.56 and 102.06 Mya in *G. hirsutum* (Table S4). Furthermore, we evaluated the Ka/Ks ratio of every duplicated gene pair to examine their rates of molecular evolution. The findings revealed that most intraspecific duplicated gene pairs had ratio values between 0.05 and 0.64 (Figure S5 and Table S4), and none had molecular evolution rates of greater than 1. To explore the evolution of the *GLN* genes in the four cotton species in more detail, we evaluated their collinearity and interspecific orthologous gene pairs. Only one orthologous gene pair (Gh_A11G178900.1_vs_Ga11G2230.1) exhibited positive selection, as shown by their Ka/Ks ratio which was greater than 1 (Figure S5 and Table S4b). Most orthologous gene pairs had a Ka/Ks ratio of less than 1, indicating that segmental duplication was the main driving force for the expansion of the *GhGLN* gene family.

**Fig. 4:**
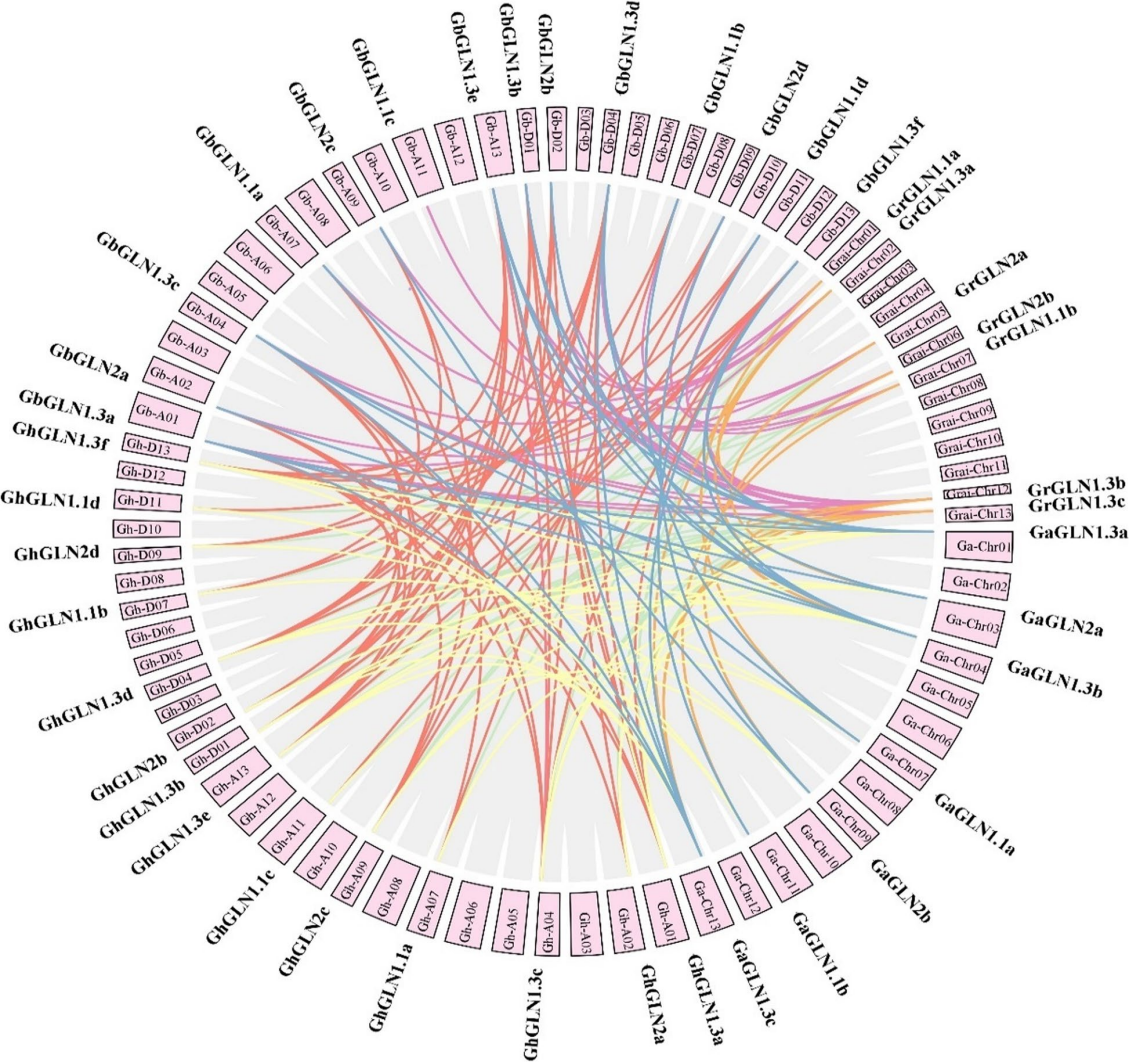
Collinearity relationship between the homologous *GLN* genes of the four cotton species. The lines in different colors connect collinear gene pairs. The chromosome numbers are shown in boxes and are denoted as GhA1–A13, GhD1‒D13, GbA1–A13, GbD1‒D13, GaChr01- Chr13 and GraiChr01- Chr13

A collinearity analysis was also performed between the sub-genomes of various cotton species (Fig. 4). Unorganized collinearity links existed between the chromosomes, with 27 collinear gene pairs between *G. arboretum* and *G. hirsutum* and 26 collinear gene pairs between *G. raimondii* and *G. hirsutum*. Considering the structural changes and lack of order of the chromosomes, the collinearity blocks were well-matched and covered most of the chromosomes. This was in line consistent with the fact that *G. hirsutum* originated through the hybridization of *G. arboreum* and *G. raimondii*, which was followed by their subsequent polyploidization [45]. There were 50 collinear gene pairings between the *G. hirsutum* and *G. barbadense,* and only one homologous gene pair was found in chromosomes Gh-A11 and Gh-D11, However, at least two homologous gene pairs were found in the other chromosomes. These four cotton species were found to have a close evolutionary relationship, although their chromosomal structures underwent major changes. TBtools were used to generate the Circos map and show the synteny of the *GLN* genes in *G. hirsutum* (Figure S6). There were 14 *GhGLN*s evenly distributed throughout 14 chromosomes of *G. hirsutum*. A complex collinearity relationship existed between the 14 *GhGLN*s, as shown in Figure S6. Each *GhGLN* in the At sub-genome matched at least one *GhGLN* in the Dt sub-genome. However, only *GhGLN1.1b* corresponded to *GhGLN1.1a*, and *GhGLN1.1d* to *GhGLN1.1c*. Five corresponding genes matched several *GhGLN*s across the entire genome; For example, *GhGLN1.3b, GhGLN1.3d, GhGLN1.3f, GhGLN1.3a, GhGLN1.3c* and *GhGLN1.3e* matched one another.

### Expression pattern analysis of *GhGLN1*s

**Fig. 5:**
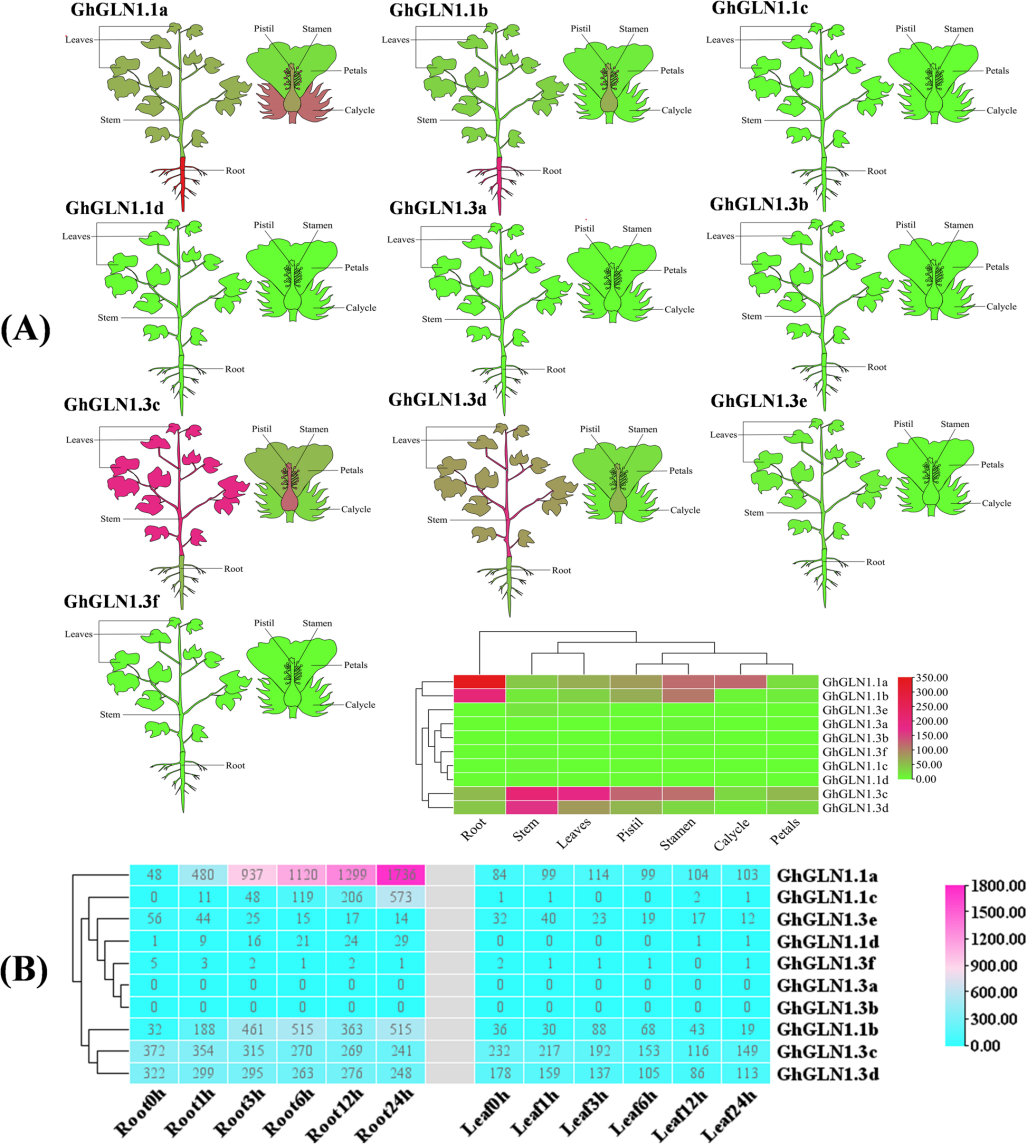
The pattern of expression the *GhGLN*1 family genes. **A** The differential expression of 10 *GhGLN1* genes in different tissues. **B** The level of expression of the *GhGLN1* genes in the roots and leaves after the N induction treatment by qRT-PCR. The mean value of expression value was calculated from three independent biological replicates. The raw data of the relative value of expression is provided in TableS6. qRT-PCR, quantitative real-time reverse-transcription PCR

Heatmaps of the expression levels of 10 *GhGLN1* genes were created to further investigate the functions of the *GhGLN* family genes. We found that four genes (*GhGLN1.1a*, *GhGLN1.1b*, *GhGLN1.3c* and *GhGLN1.3d*) were highly expressed in the roots, stems and leaves of *G. hirsutum*. The expression levels of *GhGLN1.1a* and *GhGLN1.1b* were higher in the roots, while those of *GhGLN1.3c and GhGLN1 0.3d* were more highly expressed in the stems (Fig. 5A).

The changes in the levels of expression of the 10 *GhGLN1* genes were also analyzed in the roots and leaves 0 h, 1 h, 3 h, 6 h, 12 h and 24 h after the N induction treatment. The expression level of the *GhGLN1.1a* gene in the cotton roots increased gradually and stably with time under the N induction treatment. In particular, the expression level increased tenfold after 1 h, twofold after 3 h, and nearly 40 fold after 24 h of the N induction treatment. This indicated that*GhGLN1.1a* was significantly induced by N in the cotton roots. Compared with the roots, *GhGLN1.1a* was expressed at lower levels in the cotton leaves after the N induction treatment (Fig. 5B).

**Fig. 6:**
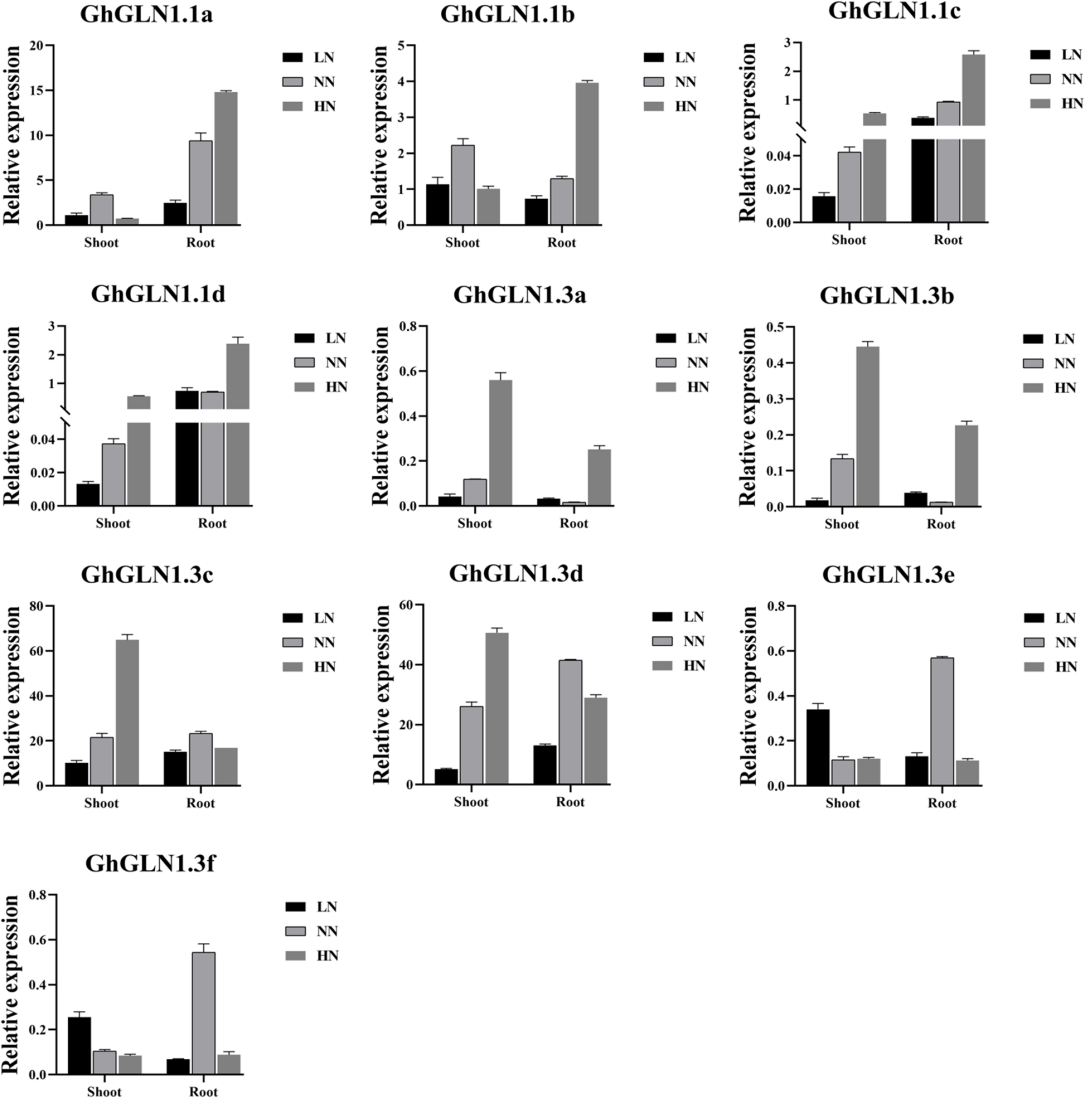
A qRT-PCR analysis of the relative expression of *GhGLN1* genes in the roots and shoots of cotton under different N concentrations. The error bars represent the mean standard deviations of three biological replicates. HN, high nitrogen; LN, low nitrogen; NN, normal nitrogen; qRT-PCR, quantitative real-time reverse-transcription PCR

To detect the expression of *GLN* gene under different N levels, a 4-week experiment was conducted, and three N treatments were set up, namely low N (LN), normal N (NN), high N (HN). After 4 weeks of growth under the N treatments, root and shoot samples were collected to measure the expression levels of the 10 *GhGLN1* genes using qRT-PCR. The levels of expression *GhGLN1.3a*, *GhGLN1.3b*, *GhGLN1.3e* and *GhGLN1.3f* genes in the roots and shoots were very low under the three N treatments. The genes *GhGLN1.3c* and *GhGLN1.3d* exhibited high expression levels in both roots and shoots across the three N treatments, with their expression levels positively correlating with N concentration in shoots. Conversely, with rising N concentrations, the expression levels of *GhGLN1.1a, GhGLN1.1b, GhGLN1.1c, and GhGLN1.1d* increased in roots, with *GhGLN1.1a* showing the most pronounced increase in expression. In the roots, the expression of *GhGLN1.1a* was 2.47 under the LN treatment and increased to 14.78 under the HN treatment, representing a six-fold rise in expression level for this gene (Fig. 6). Among the three N concentration treatments, the expression changes of the other nine genes in roots were not as pronounced as the increase observed in *GhGLN1.1a* expression, suggesting that *GhGLN1.1a* in roots is particularly sensitive to N treatments.

### Silencing of *GhGLN1.1a*

**Fig. 7:**
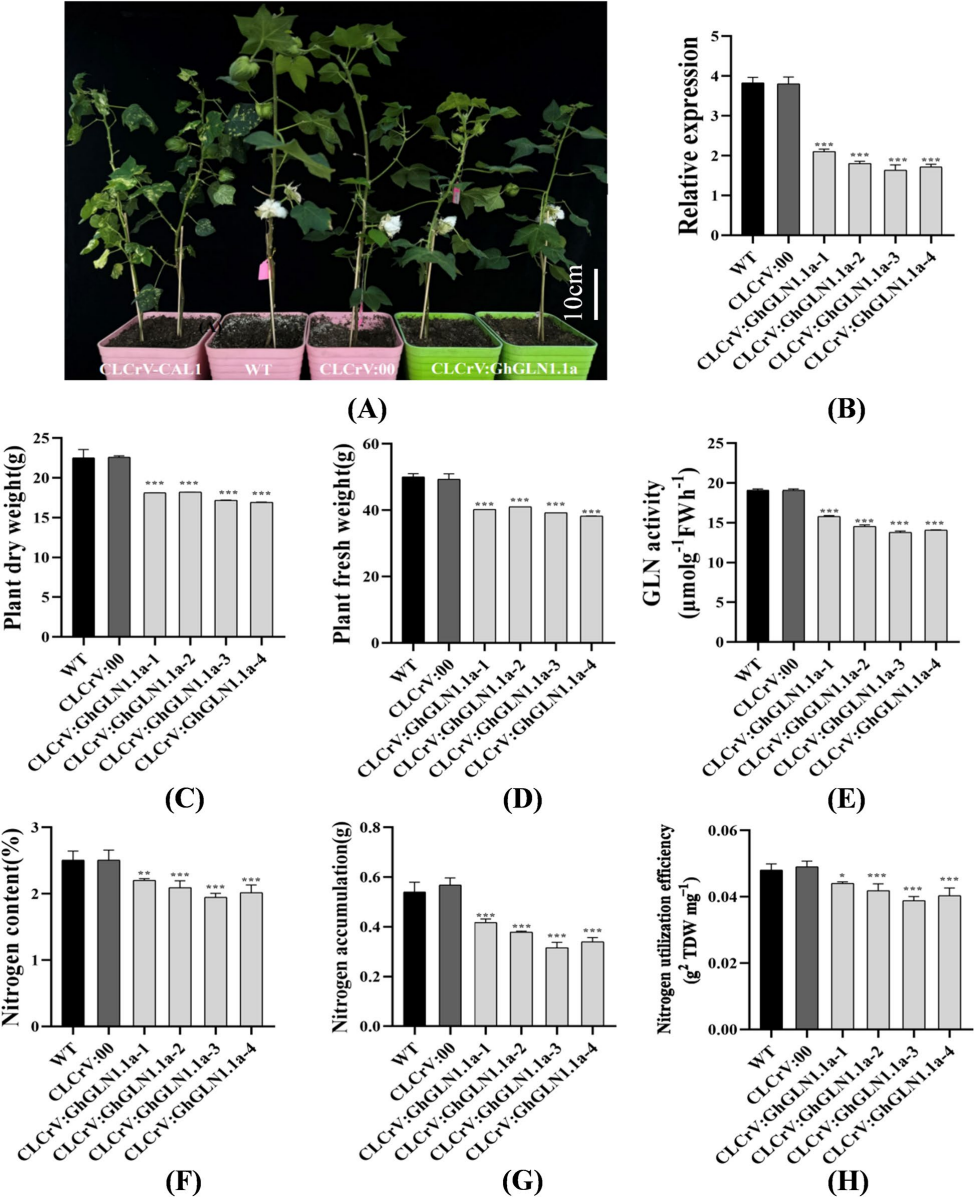
Silencing of the *GhGLN1.1a* gene by VIGS and plant growth and analyses of nitrogen use efficiency in cotton plants. **A** The phenotype of the control and gene-silenced plants. The gene-silenced plants have albino phenotypes. “CLCrV-CAL1”, chlorophyll-deficient plants; “WT”, the wild-type plants; “CLCrV:00”, the plants carrying the CLCrV empty vector; “CLCrV: *GhGLN1.1a*”, the *GhGLN1.1a*-silenced plants. **B** Relative expression level. **C** Plant dry weight. **D** Plant fresh weight. **E** Glutamine synthetase (*GLN*) activity. **F** N content of the leaves. **G** Accumulation of N in the leaves. **H** N use efficiency (NUE). VIGS, virus-induced gene sequency. Error bars show the standard deviations of the three replicate trials (*/**/*** denote significant differences between the empty vector and *GhGLN1.1a*-silenced plants, where * indicates *p* < 0.05, ** indicates *p* < 0.01 and *** suggests *p* < 0.001)

The cotton seedlings were transformed with the CLCrV-CLA1, CLCrV: 00 and CLCrV: *GhGLN1.1a* vectors. Subsequently, the efficacy of *GhGLN1.1a* silencing during cotton boll opening was assessed using qRT-PCR. The gene silencing induced by CLCrV: *GhGLN1.1a* was effective in the infiltrated leaves (Fig. 7A-B). The plant dry weight, plant fresh weight, *GLN* activity, N contents, N accumulation, and the NUE of the CLCrV:*GhGLN1.1a*-silenced plants were significantly lower compared with those of the CLCrV: 00 plants (Fig. 7C-H). This confirmed that the inactivation of *GhGLN1.1a* affected the activity of *GLN*, N accumulation, and NUE.

## Discussion

Glutamine synthetase (*GLN*) is an essential enzyme for the metabolism of N and the assimilation and remobilization of ammonium. *GLN* are complicated owing to their existence as two isoenzymes, one in the chloroplast and the other in the cytosol, and the fact that the cytosolic one has many isoforms. The six *GLN* genes of *A. thaliana* are anticipated to have various functions depending on the plant organs and the N availability in the soil. A multigenic family encodes the multiple isoforms [21]. Similarly, the functions of five *GLN1* genes in maize depend on the N levels [46]. Nevertheless, *GLN* genes in cotton are still unclear, particularly under different N levels. This study purposed to analyze the expression of the *GhGLN* genes under different N levels and identify the entire *GLN* gene family in cotton.

*GLN* isoforms have been examined in various plants, including soybeans [47], potatoes [48] and tomato [37], thus, establishing a crucial foundation to study the functional *GLN* isoforms. However, the *GLN* family members and their functions have not yet been reported in cotton. This study employed bioinformatics analysis to investigate the structure and function of *GLN* family members in cotton. For the analysis of cotton *GLN* genes' functions, we utilized 42 identified *GLN* genes identified as potential candidates. These *GLN* genes were categorized into three lineages and named based on phylogenetic trees constructed using their amino acid sequences. A portion of the functional conservation of the proteins was represented by exon–intron and protein structural conservation. An analysis of gene structure and conserved motifs showed that the *GLNs* has a higher structural consistency. Most *GLNs* comprised 10 identical motifs, and no subfamily-specific motifs were found. We also found that other genes with close biological relationships in the same subfamily had the same motif eliminated. Additionally, the *GLNs* from the same subfamily shared similar gene structures and motifs, which indicated that the conserved motifs and gene structural changes may have greatly influenced the functional evolution of the *GLN* genes in cotton. The similar structures and patterns of the *GLN* genes also indicated the possibility of interactions between genes from the same subfamily.

PlantCARE is a database of the plant cis-regulatory elements, it has abundant important regulatory sequences, including cis-regulatory elements, enhancers and suppressors, and can provide important information to predict the molecular regulatory mechanism of genes [21]. Cis-elements and trans-acting factors can control the levels of expression of the genes by interacting with the transcription factors (TFs) of the targeted genes [49]. As a result, the expression of the targeted genes is differentially regulated by the cis-acting regions. An analysis of the promoter regions revealed that each member of the *GhGLN1* gene family had a different number and arrangement of regulatory components. Each *GhGLN1* gene may be subject to complex regulation, depending on the amount and types of regulatory elements in the *GhGLN1* gene family. This indicates that the roles of these genes are not duplicated. This study used the PlantCARE database to analyze the 2000 bp nucleotide sequences upstream of ATG of the 10 *GhGLN1* genes. The results showed that the 5’ terminal promoter region of the *GhGLN1* gene contained binding elements specific to auxin response, low-temperature response, plant hormone response, and MYB TFs. An analysis of the gene sequences showed that most of the 10 members contained MYB binding sites, which might explain the regulation of MYB TFs in the *GhGLN1* gene family (Fig. 2). EI- Kereamy et al. [22] found that the content of amino acids increased significantly in transgenic rice that overexpressed *OsMYB55* because of the expression levels of synthetases of some amino acids, including *OsGS1. 2* genes were changed, which indicated that e *OsMYB55* could regulate the expression of *OsGS1. 2* genes. In addition, our results showed that there were some important regulatory elements in the 2000 bp sequence upstream of the ATG of the *GhGLN1* genes, and these included response elements specific to ABA, MeJA, SA, auxin, gibberellin and other response elements. Currently, there are few reports on the regulation of *GLN* genes by these response elements, thus necessitating further studies.

Polyploidy is a key factor in evolution and a crucial mechanism for the diversification of plants [50]. The hybridization of the *G. raimondii*-like D-genome ancestor (D5) and the *G. arboreum*-like A-genome ancestor (A2) yielded the all-tetraploid cotton (*G. hirsutum*), which then underwent chromosomal doubling [51]. As previously shown study, the At and Dt subgenomes evolved differently, with structural rearrangements and gene deletions occurring more frequently in the At subgenome [42]. However, we found that no *GLN* genes were lost in the At or the Dt subgenomes, and the *GLN* genes of the tetraploid cotton were identical to those in the diploid ones. It has been demonstrated that duplicated gene pairs regularly develop through various mechanisms, including pseudogenization, neo-functionalization and sub-functionalization [52]. These provide a testable hypothesis that the neo-functionalized gene copies exhibit positive selection (Ka/Ks > 1), whereas the sub-functionalized gene copies are susceptible to purifying selection (Ka/Ks = 1) [53]. In this study, the Ka/Ks ratio of the most duplicated gene pairs was less than one, which suggested that these genes mainly primarily their original functions and were subject to purifying selection pressure during evolution. Only one orthologous gene pair between *G. arboretum* and *G. hirsutum* (At) had the Ka/Ks ratio greater than one. These findings suggest that throughout the *GLN* genes of At sub-genomes may have undergone more positive selection than the Dt sub-genomes during the development of tetraploids from diploids.

Using pre-existing RNA-seq and qRT-PCR data, we constructed heat maps illustrating the expression levels of the 10 *GhGLN1* genes. This allowed for a more detailed investigation into the functions of *GLN* family genes. The results showed that the *GhGLN1.1a* gene was highly and specifically expressed in the roots, and its expression in the cotton roots was significantly induced by N induction. Plants respond to abiotic stresses by activating numerous molecular, cellular and physiological mechanisms, which negatively impact their growth and development. Most *GhGLN1* genes are associated with various environmental conditions, including cold, heat, salt and drought, and their patterns of expression depend on their responses to abiotic stimuli. The expression levels of 10 *GhGLN1* genes were examined under cold, heat, saline and drought abiotic stresses. The expression of the *GhGLN1.1a* gene was significantly increased after 12 h of cold, heat, salt and drought stress, which indicating that the gene may have some regulatory roles under cold, heat, salt and drought stresses.

Since the 3–5 *GLN1* members in plants show specificity in their expression in tissues, it is important to study the biological functions of different *GLN1* genes to clarify the mechanism of the development of specific tissues or organs of plants [36]. The qRT-PCR results in this study showed that *GhGLN1.1a*, *GhGLN1.1b*, *GhGLN1.1c* and *GhGLN1.1d* were highly expressed and specifically expressed in the cotton roots. The qRT-PCR data analysis also showed that the *GhGLN1.1a* gene was specifically expressed in cotton roots and significantly induced by the N induction treatment. Therefore, *GhGLN1.1a* was selected as a representative gene for further functional analysis. Previous research showed that overexpression of the *GLN1* gene can enhance the activity of *GLN* and significantly impact key agricultural variables, such as plant biomass and yield. Early studies in pine trees found that the growth characteristics of transgenic strains were improved by ectopic expression of the *GS1a* gene in pine trees under greenhouse and field conditions [41]. Studies in maize, wheat, rice, and *A. thaliana* also showed that the *GLN1* gene greatly increased the grain yield. Thus, the phenotypic performance of various plants that overexpressed *GLN1* homologous genes is inconsistent. This indicates that the upstream or downstream sequence of the *GLN1* gene may affect the *GLN* activity, thereby affecting its biological function to some extent. Thus, it is necessary to study the molecular regulatory mechanism of the *GLN* gene to improve the NUE of plants [42, 43]. In this study, *GhGLN1.1a* was silenced using VIGS, and the silencing efficiency was approximately 50% during the spit stage, at which the *GLN* activity and NUE were significantly reduced compared with the wild-type plants. However, owing to the high homology of the *GLN* isoenzymes and the lack of effective specific antibodies, the localization, expression and functional analyses of *GLN* isoenzymes are mostly conducted at the transcription level. Moreover, the expression of *GLN* is also strictly regulated at the transcription, post-transcriptional and post-translational stages, which indicates that studying *GLN* at the transcriptional level alone does not fully reveal its role in N assimilation.

*GLN* is regulated at multiple levels, including during the transcription of *GLN* genes, polyadenylation for mRNA stability, peptide synthesis, post-translational modifications and protein transport. In addition, many other compounds affect *GLN* activity; for example, Takashi showed that the ACR11 protein promotes *AtGLN2* activity in *A. thaliana*. Lima [42] showed that *AtGLN2* is regulated by phosphorylation and interaction with 14–3-3 proteins. *NLP7* TFs induce the expression of *GS2*. In this study, gene sequence analysis showed that the 5’ end promoter region of the *GS* gene contained elements that specifically bind the transcription factors such as MYB (v-myb avian myeloblastosis viral oncogene homolog) and Dof (DNA binding with one finger). Recently, El-Kereamy et al. found that the amino acid content in the rice *trans-OsMYB55* gene had increased significantly owing to the altered expression of the synthases for some amino acids, including *OsGS1:2*, which indicated that *OsMYB55* can regulate the expression of *OsGS1:2* [48]. The initiator regions of the six *GS* genes in maize contained MYB binding sites, *ZmGS1-2* and *ZmGS1-2* genes had five MYB binding sites, *ZmGS1-2* and *ZmGS1-5* contained three each, while *ZmGS1-3* and *ZmGS2* contained two and one, respectively. The existence of multiple MYB binding sites also suggests their complex regulatory mechanisms [48]. Studies on pine Dof5 showed that Dof5 transcription factors can concurrently regulate the expression of *GS1a* and *GS1b* to promote the expression of *GS1b* while inhibiting the transcription of *GS1a*, thereby regulating the spatial distribution of GS1 isoenzymes in pine trees [49]. In addition, some members of the *LBD* (Lateral organ boundaries domain) gene family regulate N metabolism [50]. Rubin et al. found that the expression of some N metabolism genes and the content of amino acids (glutamate) were significantly reduced in the *A. thaliana* plants that overexpressed three *LBD* genes, including the *GLN* gene [51]. Therefore, *GhGLN1* was studied at the post-transcriptional and post-translational levels in subsequent experiments to fully reveal its role in N assimilation.

## Conclusions

The study identified 7, 7, 14, and 14 members of the *GLN* gene family from the genomes of *G. raimondii, G. arboreum, G. hirsutum,* and *G. barbadense*, respectively. These *GLN* genes were classified into three lineages based on their phylogenetic relationships, as well as their possession of similar gene structures and motifs. Genome localization revealed one *GLN* gene per chromosome, predominantly located at the distal ends. Whole-genome duplication significantly influenced the expansion of the *GLN* family in cotton. Notably, *GhGLN1.1a* exhibited the highest expression levels under various abiotic stresses and harbored regulatory elements responsive to anaerobic conditions and phytohormones. qRT-PCR analysis revealed root-specific expression of *GhGLN1.1a*, particularly sensitive to N induction, suggesting its significance for further functional analysis. VIGS experiments demonstrated that inactivation of *GhGLN1.1a* affected N accumulation and NUE. These findings provide a foundation for future research on the functionality of *GLN* proteins.

### Supplementary Information

**Supplementary Material 1.**

**Supplementary Material 2.**

**Supplementary Material 3.**

**Supplementary Material 4.**

**Supplementary Materila 5.**

**Supplementary Material 6.**

**Supplementary Material 7.**

**Supplementary Material 8.**

**Supplementary Material 9.**

**Supplementary Material 10.**

**Supplementary Material 11.**

**Supplementary Material 12.**

## Data Availability

The datasets supporting the conclusions of this article are included within the article and its additional files.
