# Supplementary figures and images for: Systematic characterization of Gossypium GLN family genes reveals a potential function of GhGLN1.1a regulates nitrogen use efficiency in cotton
Source: BMC Plant Biol. 2024 Apr 23;24:313. doi: 10.1186/s12870-024-04990-0 (PMC11036627; doi:10.1186/s12870-024-04990-0)

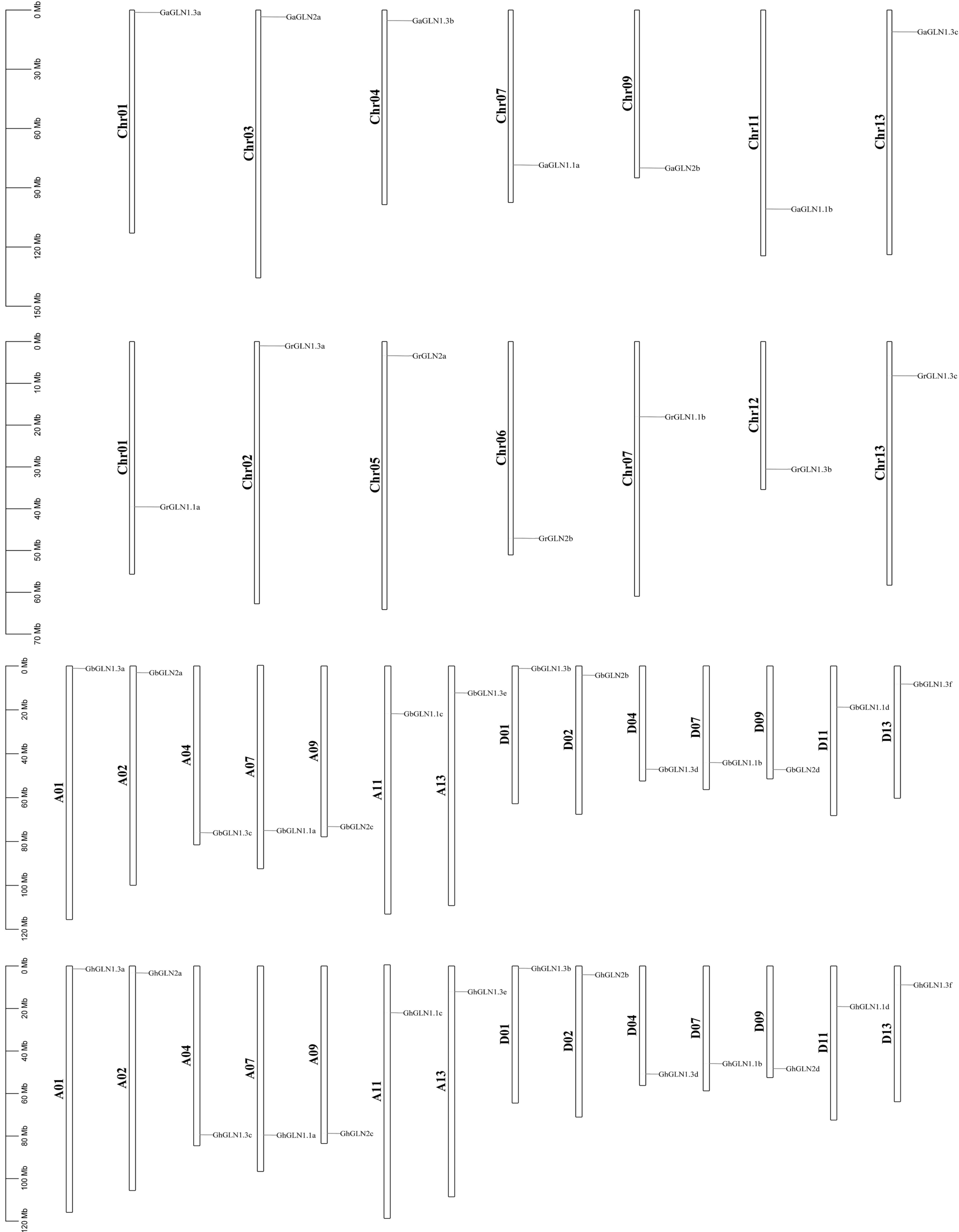

Figure S1 Distribution of *GLN* genes in the chromosomes of four cotton species

Supplement: Supplementary file 7 — Supplementary Material 7. [file 12870_2024_4990_MOESM7_ESM.pdf]

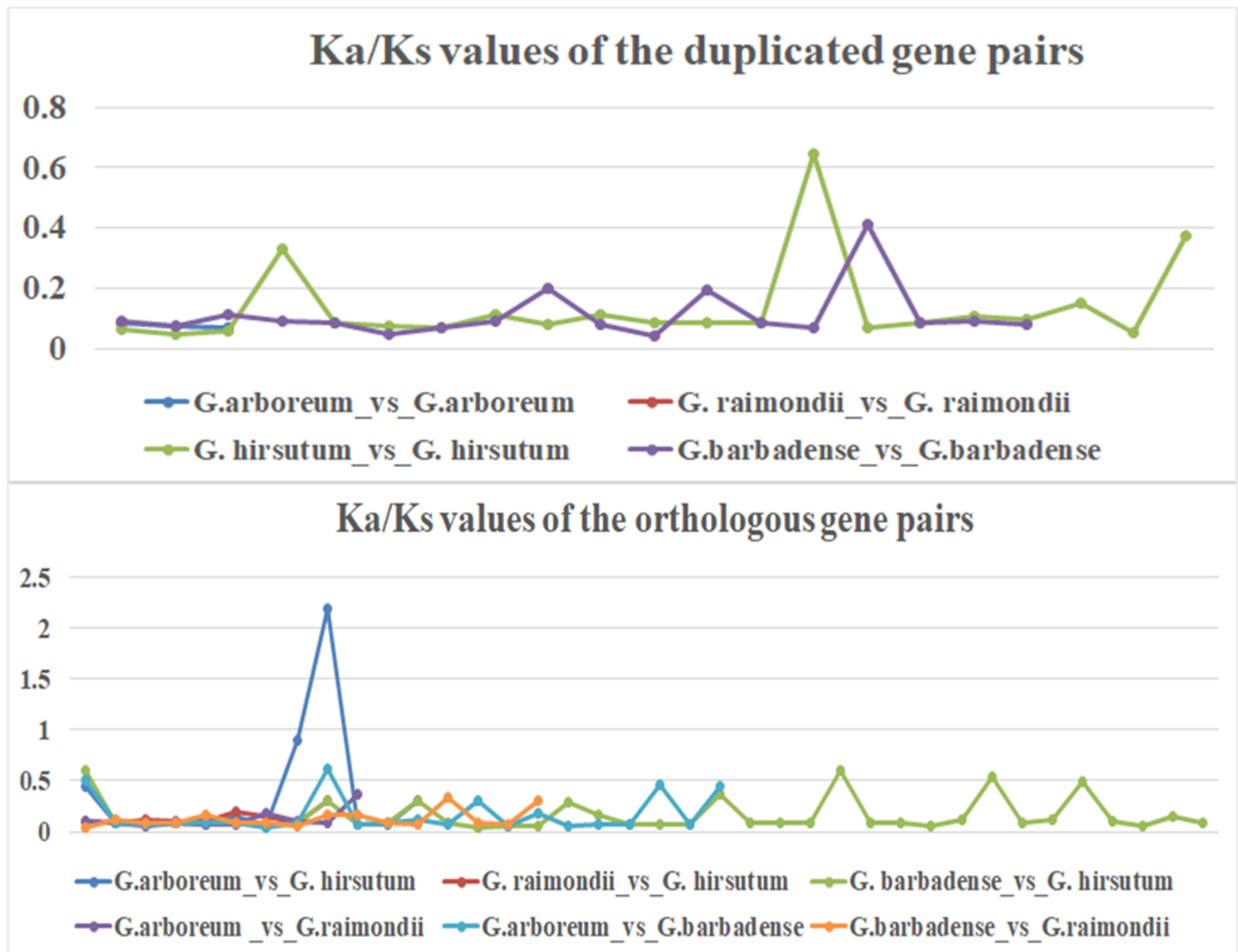

**Figure S5 The synonymous and non-Synonymous ratio of *GLN* gene family in four cotton species**

Supplement: Supplementary file 11 — Supplementary Material 11. [file 12870_2024_4990_MOESM11_ESM.pdf]
