# Supplementary material for: Systematic characterization of Gossypium GLN family genes reveals a potential function of GhGLN1.1a regulates nitrogen use efficiency in cotton
Source: BMC Plant Biol. 2024 Apr 23;24:313. doi: 10.1186/s12870-024-04990-0 (PMC11036627; doi:10.1186/s12870-024-04990-0)
Supplement: Supplementary file 8 — Supplementary Material 8. [file 12870_2024_4990_MOESM8_ESM.pdf]

	Logo	E-value	Sites	Width
1.	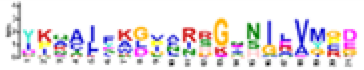	5.4e-1746	194	21
2.	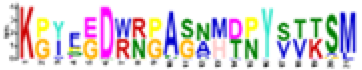	9.7e-1444	93	21
3.	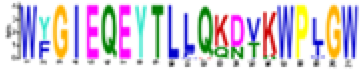	1.3e-931	48	21
4.	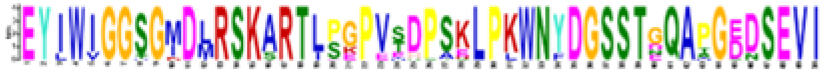	4.8e-1730	40	50
5.	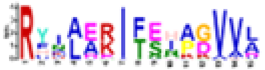	3.3e-809	95	15
6.	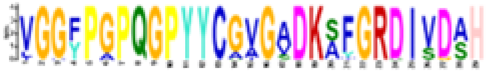	1.5e-1221	48	29
7.	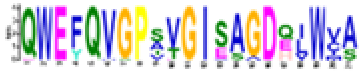	6.5e-808	48	21
8.	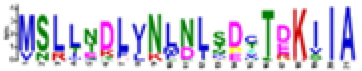	4.1e-500	39	21
9.	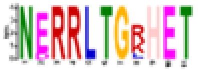	2.0e-428	48	11
10.	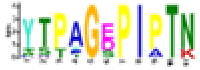	8.0e-393	60	11
11.	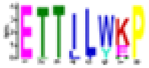	6.6e-213	44	8
12.	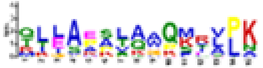	3.7e-169	41	15
13.	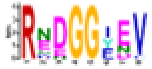	1.4e-161	48	8
14.	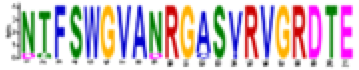	3.1e-115	9	21
15.	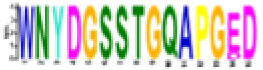	1.2e-050	7	15

Figure S2. Sequence logo of the GhGLNs conserved-domain
