# Supplementary material for: Systematic characterization of Gossypium GLN family genes reveals a potential function of GhGLN1.1a regulates nitrogen use efficiency in cotton
Source: BMC Plant Biol. 2024 Apr 23;24:313. doi: 10.1186/s12870-024-04990-0 (PMC11036627; doi:10.1186/s12870-024-04990-0)
Supplement: Supplementary file 9 — Supplementary Material 9. [file 12870_2024_4990_MOESM9_ESM.pdf]

	AtGLN1.1	GhGLN1.1c	GhGLN1.1d	GhGLN1.1a	GhGLN1.1b	AtGLN1.2	AtGLN1.3	GhGLN1.3a	GhGLN1.3b	GhGLN1.3c	GhGLN1.3d	AtGLN1.5	GhGLN1.3e	GhGLN1.3f	AtGLN1.4
AtGLN1.1	***	86.5	88.2	**90.4**	89.9	**91.9**	86.2	80.9	80.1	87.9	84	79.5	82	80.6	88.5
GhGLN1.1c	14.9	***	**97.8**	**93**	**92.7**	85.1	84.7	83.1	82	87.9	83	81	83.1	81.7	86
GhGLN1.1d	12.9	2.3	***	**94.7**	**94.4**	86	86.2	84.6	83.4	89.3	84.6	82.4	84.3	82.9	87.6
GhGLN1.1a	10.2	7.4	5.5	***	**99.2**	87.4	88.1	84.8	84	**91**	86.5	82.1	85.1	84	88.2
GhGLN1.1b	10.9	7.7	5.8	0.8	***	87.1	87.6	84.6	83.7	**90.7**	86.2	81.8	84.8	83.7	88.5
AtGLN1.2	8.6	16.6	15.6	13.9	14.2	***	83.9	81.2	80.1	87.6	83.6	79.3	80.6	80.1	86.2
AtGLN1.3	15.3	17.1	15.3	12.9	13.6	18.2	***	84.7	83.9	87.3	82	83.2	81.6	80.8	83.3
GhGLN1.3a	22.1	19.1	17.3	17	17.3	21.7	17.1	***	**98.6**	87.4	82.4	79.3	83.4	82	82.3
GhGLN1.3b	23.2	20.6	18.8	18.1	18.4	23.2	18.2	1.4	***	86.8	82.1	78.7	82.9	82	81.5
GhGLN1.3c	13.2	13.2	11.5	9.6	9.9	13.5	14	13.9	14.6	***	**93.7**	82.4	89	87.6	87.9
GhGLN1.3d	18.1	19.3	17.3	15	15.3	18.5	20.6	20.1	20.5	6.6	***	77.8	83.3	82.4	84.3
AtGLN1.5	23.9	22	20.1	20.5	20.9	24.3	19	24.3	25.1	20.1	26.4	***	78.7	77.6	80.1
GhGLN1.3e	20.6	19.1	17.7	16.6	17	22.5	21.1	18.8	19.5	11.9	18.9	25.1	***	**97.5**	81.7
GhGLN1.3f	22.5	21	19.5	18.1	18.4	23.2	22.2	20.6	20.6	13.5	20.1	26.7	2.6	***	81.2
AtGLN1.4	12.5	15.6	13.5	12.9	12.5	15.2	18.9	20.2	21.4	13.2	17.7	23.2	21	21.7	***

Figure S3 GhGLN1 proteins and AtGLN1 proteins identities. The percentage identity between GhGLN1 translated protein sequences, and between AtGLN1 translated protein sequences. Light grey indicates percentage identity between A. thaliana and G.hirsutum genes. Dark grey indicates identity between G.hirsutum genes. Identities >90% are in bold.
