# Supplementary material for: Systematic characterization of Gossypium GLN family genes reveals a potential function of GhGLN1.1a regulates nitrogen use efficiency in cotton
Source: BMC Plant Biol. 2024 Apr 23;24:313. doi: 10.1186/s12870-024-04990-0 (PMC11036627; doi:10.1186/s12870-024-04990-0)
Supplement: Supplementary file 10 — Supplementary Material 10. [file 12870_2024_4990_MOESM10_ESM.pdf]

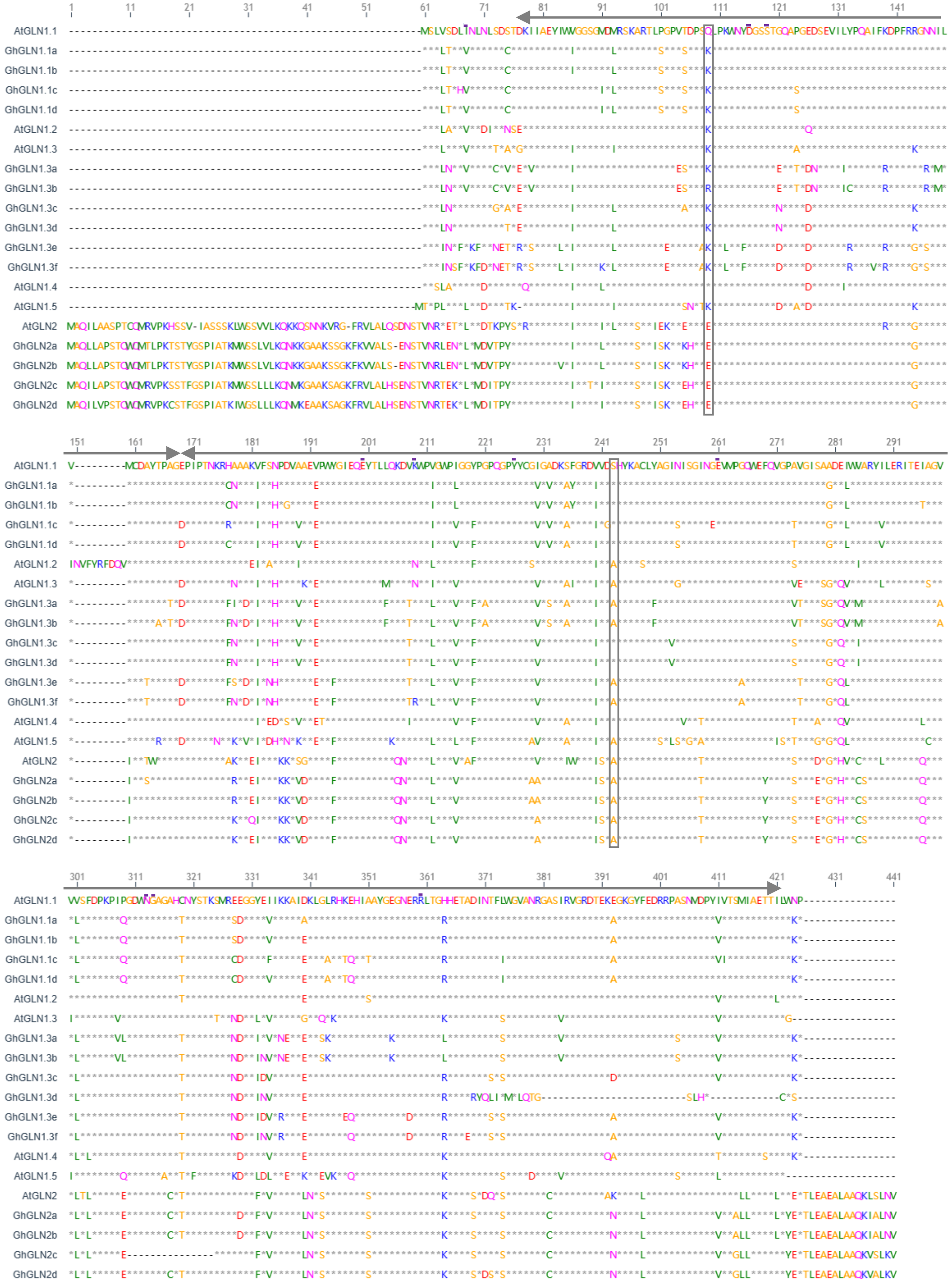

Figure S4. Alignment of Gossypium hirsutum and Arabidopsis thaliana GLN proteins. Protein sequences were identified using DNA coding sequences and then Clustal was used to align them. The ammonium/glutamate-binding pocket residues are indicated by a short purple line. Residues associated with ammonium affinity characteristics are shown in boxes . Conserved domains are shown by arrows (1) pfam 03951 Gln-synt_N glutamine synthetase bet-Gasp domain; and (2) pfam 00120 gln-synt_C catalytic domain. The physical and chemical characteristics of residues determine their color.
