# Supplementary material for: Systematic characterization of Gossypium GLN family genes reveals a potential function of GhGLN1.1a regulates nitrogen use efficiency in cotton
Source: BMC Plant Biol. 2024 Apr 23;24:313. doi: 10.1186/s12870-024-04990-0 (PMC11036627; doi:10.1186/s12870-024-04990-0)
Supplement: Supplementary file 12 — Supplementary Material 12. [file 12870_2024_4990_MOESM12_ESM.pdf]

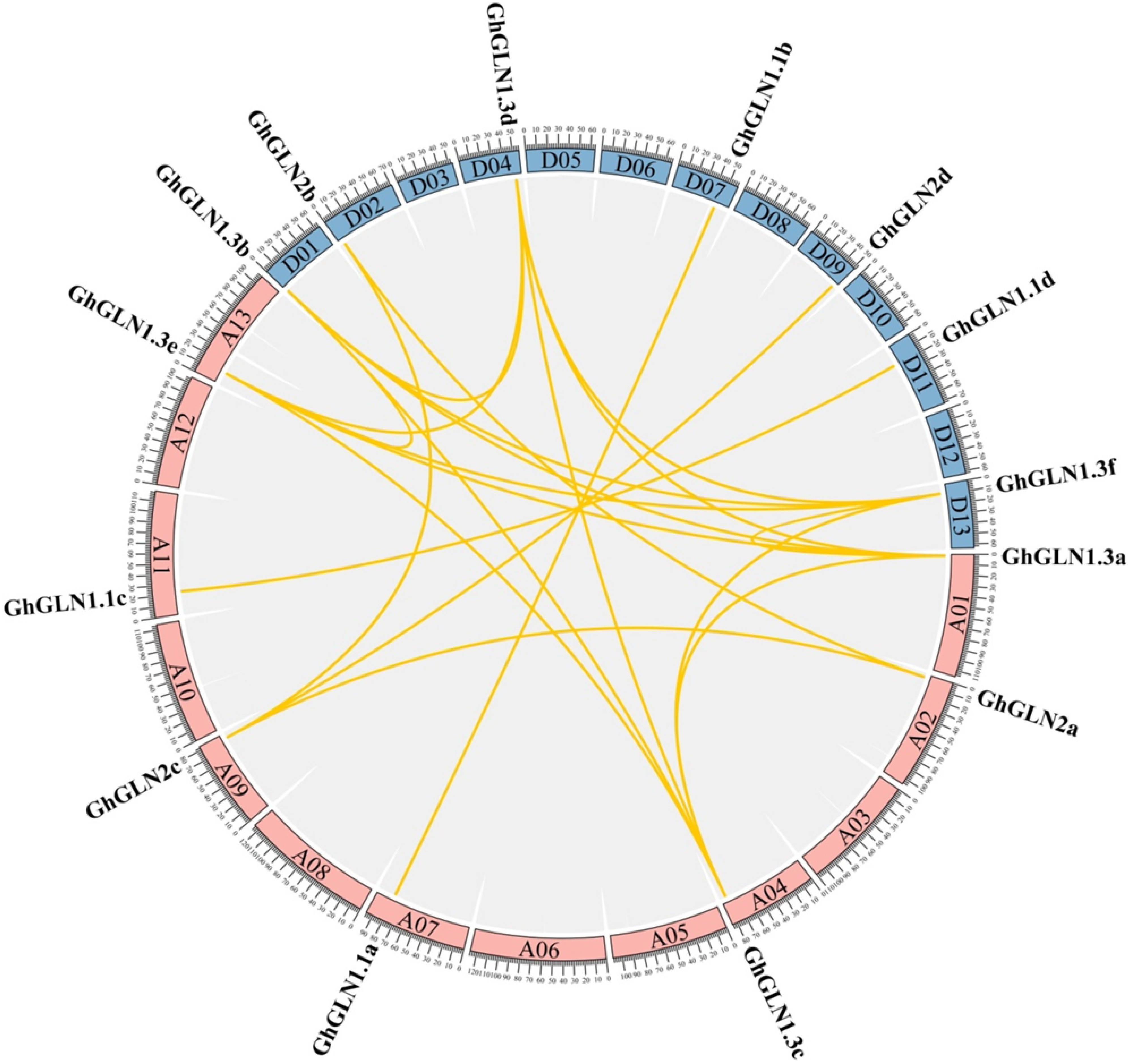

Figure S6 Chromosomal distributions and collinearity relationship of the identified *GhGLNs*. The collinear gene pairs are connected by yellow lines. The chromosome numbers are indicated in boxes and represented as A1–A13 and D1–D13.
